# Post-transplant lymphoproliferative disease

**DOI:** 10.1007/s00467-007-0582-3

**Published:** 2009-04-01

**Authors:** Vikas R. Dharnidharka, Carlos E. Araya

**Affiliations:** Division of Pediatric Nephrology, University of Florida College of Medicine, PO Box 100296, 1600 SW Archer Road, Gainesville, FL 32610-0296 USA

**Keywords:** Post-transplant lymphoproliferative disease, Pediatrics, Kidney

## Abstract

Post-transplant lymphoproliferative disease (PTLD) emerged in the mid-1990s as a major graft- and life-threatening complication of pediatric kidney transplantation. This condition, usually involving uncontrolled B lymphocyte proliferation, straddles the border between infection and malignancy, since Epstein–Barr virus (EBV) is intimately associated with the pathogenesis. PTLD is seen more in younger children (more likely to be EBV seronegative), Caucasian race, and in association with the more potent immunosuppression drugs. The clinical presentation typically involves multiple enlarged lymph nodes but varies based on localization of the lymphadenopathy. The diagnosis is based primarily on histopathological features. Treatment strategies include reduction of immunosuppression, use of anti-B cell antibodies, infusion of EBV-specific cytotoxic T lymphocytes, and chemotherapy. Many different strategies have been tried to prevent PTLD, ranging from serial EBV viral load monitoring and pre-emptive immunosuppression reduction to anti-viral prophylaxis. None of the major treatment or prevention strategies has been subject to randomized clinical trials, so their relative efficacy is still unknown. PTLD remains a risk factor for graft loss, though re-transplants have not, to date, been associated with repeat PTLD.

## Introduction

PTLD is a major graft- and life-threatening complication of solid organ transplantation. This condition is best defined as an uncontrolled proliferation of lymphocytes within the context of post-transplant immunosuppression. In children, most of the proliferating lymphocytes are of B cell lineage and are driven by Epstein–Barr virus infection [1]. Sometimes, the proliferations are reversible by reduction of immunosuppression, thus distinguishing PTLD from true malignancy. At other times the severe forms of PTLD are indistinguishable from frank lymphoma. Previously thought to be rare in children receiving kidney transplants, the cumulative incidence of PTLD in this population kept rising through the mid-1990s and early part of this decade [2]. The emergence of Epstein–Barr virus (EBV)–PTLD was part of a sequence of emerging viral infections in transplantation, starting with cytomegalovirus (CMV), followed by EBV and later followed by BK virus. This teaching article focuses on PTLD after pediatric kidney transplantation. Several excellent reviews have covered PTLD across different organ and tissue transplants [1, 3–8].

## Pathophysiology

The pathophysiology of PTLD has been described [1, 4, 8]. EBV is a DNA virus of the gamma herpes family. Most immunocompetent individuals acquire subclinical infection at some point prior to adulthood. The virus establishes a long-lived latency in reticuloendothelial cells, similar to another herpes virus cytomegalovirus. B lymphocytes are an important reservoir for EBV. Left unchecked, the virus will insert its own genome into the B cell and induce uncontrolled B cell proliferation. However, immunocompetent individuals can keep the infected B cells in check through EBV-specific CD8+ cytotoxic T lymphocytes (CTLs). When transplant recipients receive immunosuppression to prevent rejection, an important consequence of such non-specific immunosuppression is the inhibition of the EBV-specific CTLs. Thus, in this setting, EBV-infected B cells may proliferate unchecked. This proliferation is particularly marked when the transplant recipient is EBV seronaive and acquires a primary infection when immunosuppressed.

## Epidemiology of PTLD

The highest risk of PTLD is in the EBV seronegative recipient who receives a kidney allograft from an EBV seropositive donor. Multiple studies have shown that the risk of PTLD in such cases is increased threefold to 24-fold [5, 9–13]. CMV co-infection can further add to the risk [13].

PTLD rates vary with the organ transplanted. Thoracic (heart, lung or combined) and intestinal transplants have the highest rates of PTLD, typically in the 5–10% range [5, 14]. Pediatric kidney transplants were previously associated with PTLD rates of < 1%, but these rates have climbed and are now in the 2–4% range at 3–5 years after transplantation [2, 14, 15]. Prospective studies have mirrored this trend. The IN01 study was the first National Institutes of Health (NIH)-sponsored multi-centered clinical trial in pediatric kidney transplantation in the USA, conducted between 1994 and 1999. In that study, the PTLD rates were 3.4% in the OKT3 induction arm and 2.1% in the cyclosporine A (CsA) induction arm, with a mean follow-up time of 44 months for all study participants [16]. In contrast, the next NIH-sponsored study, the SW01 trial of steroid withdrawal in pediatric kidney transplant recipients conducted between 1999 and 2004, had an overall PTLD rate of 6% in both arms, leading to early termination of that study [17]. In comparison to kidney allografts, PTLD rates are 8% by 5 years after transplantation in the Pediatric Heart Transplant Study [5]. The most recent national data from pediatric liver transplants, from the United Network for Organ Sharing (UNOS) and SPLIT liver transplant registries, indicate a 2.4% cumulative incidence of PTLD [14].

PTLD was very rare in the days of two-drug immunosuppression after transplantation using azathioprine and orally administered steroids. Reports of PTLD started increasing after the introduction of cyclosporine A and then other newer immunosuppressive agents, such as induction antibodies. In general, the higher the “total intensity” of immunosuppression, the higher the risk for PTLD. Individual drugs may or may not increase the risk. For most of the typically used drugs, small single center studies cannot accumulate large enough samples to delineate a higher risk fully. In contrast, large registry databases, especially in the United States of America, can evaluate large samples but do not have EBV information, and the quality and completeness of the data are inferior to single center information. Thus, both types of studies involve a trade-off. As such, CsA, OKT3, rabbit anti-thymocyte globulin, basiliximab and daclizumab have all been associated with higher PTLD risk in at least one study [13, 18, 19], though, for each drug, there are also studies documenting no higher risk [20–22]. Cyclosporine was compared to tacrolimus by the Cochrane Review Group, and no significant differences in relative risk for PTLD were found in this meta-analysis of 30 different prospective trials [23]. Mycophenolate mofetil has not been associated with higher PTLD risk to date [2] but has been associated with higher risk for CMV and BK virus infections [24]. Sirolimus has shown some anti-EBV effects in vitro [25, 26], and a large registry study suggested lower incidence of malignancy with early use of mammalian target of rapamycin (mTOR) inhibitor [27].

## Pathology

PTLD lesions can range from mild lymphoid hyperplasia to frank lymphoma, with several grades in between [28]. Most lesions are either polymorphic or monomorphic. Additional classifications based on clonality are available, dividing lesions into polyclonal and monoclonal, the latter considered more severe. EBV staining via Epstein Barr early RNA (EBER) and latent membrane protein (LMP) help us to determine if the tumor cells contain EBV. CD20 staining of the lymphocytes helps us to decide if anti-CD20 antibody therapy will be useful.

## Clinical features

PTLD can present in a variety of ways, so a high index of suspicion must be maintained at all times [29]. The most common presentation is with lymph node enlargement, either in easily visible sites (such as cervical) or internal sites not immediately visible. In the latter case the symptoms are related to whichever internal structures have been compressed or impaired in function. Internal areas of presentation include the gut, bone marrow or central nervous system. Mass effects in these areas can present as vomiting/diarrhea, hematological cytopenias, or symptoms of brain tumors. The latter has the worst prognosis amongst all presentations. From North American Pediatric Renal Transplant Cooperative Study (NAPRTCS) data, lymph node involvement was the most common presentation, followed by abdomen, kidney allograft and central nervous system (Table 1). PTLD can involve the allograft itself and may rarely present as fever with no localizing features.

**Table 1:** Distribution of PTLD by anatomic location in pediatric kidney transplant recipients (data from NAPRTCS)

Site of involvement	Percentage of total
Lymph node	33
Abdomen	29
Kidney allograft	11
Central nervous system	11
Other	16

In children receiving kidney transplants, unlike in adults, most cases of PTLD occur in the first year after transplantation, when immunosuppression is most intense and chances of EBV primary infection are higher in the D+/R- mismatch. Early PTLD cases are almost always likely to be EBV-driven and of B cell lineage. The majority of late PTLD cases are also EBV driven and of B cell lineage, but the proportion of non-EBV or T cell PTLD is higher in late PTLDs. In addition to central nervous system involvement, other poor prognostic factors include monoclonal lesions, OKT3 use, elevated lactate dehydrogenase levels, and high tumor staging scores [30–34].

## Diagnosis

The diagnosis is essentially based on histopathology, so a tissue specimen is usually needed. High peripheral blood EBV polymerase chain reaction (PCR) loads can support a diagnosis of EBV-PTLD but are not diagnostic themselves. Similarly, imaging techniques such as computed tomography (CT) or positron emission tomography (PET) [35] can help localize and stage the tumor but are not diagnostic. Oncologists can help with this staging. The differential diagnosis can include Bartonella infection [36], tuberculosis or reactive lymphadenopathy. EBV serology and monospot tests are not useful for diagnosis in the transplant setting.

## Treatment

Most physicians will first reduce immunosuppression once PTLD has been diagnosed. However, there is no consensus on how to reduce immunosuppression or which drugs to target first. A commonly used approach is to reduce calcineurin inhibitors to half their dose or target level and discontinue anti-metabolite agents, while continuing oral steroid treatment [37]. This strategy is revaluated quickly, usually within 1–4 weeks, to determine if additional interventions are needed. Such revaluations should include clinical reassessment, EBV PCR response if initial load was high, and may also include follow-up imaging if needed. In cases of severe, disseminated disease, discontinuation of all immunosuppression, with the exception of steroids, has been tried. Anti-CD20 monoclonal antibody therapy (rituximab) is now widely accepted as the second line therapy in CD20-positive lesions [38, 39]. Alpha interferon was used previously but has been replaced by rituximab. For more severe lesions or those that do not respond, chemotherapy with protocols used for non-Hodgkin’s lymphoma is employed, in conjunction with oncologists. Some protocols in common use will lower the dose of cyclophosphamide or combine low-dose cyclophosphamide and steroids with rituximab [40, 41]. Each of these strategies has a moderately high success rate in the appropriate situation, but no strategy is associated with 100% complete remission. There are no head-to-head randomized comparison trials to judge which therapy is more efficacious. Many centers will also use anti-viral agents concomitant with the above interventions. Typically, ganciclovir or valganciclovir are employed for this purpose, since these drugs are much more potent against EBV than acyclovir. The use of these drugs to treat or even prevent PTLD, though widespread, is considered controversial, since they affect only replicating viruses. In PTLD, most of the viral genome is in non-replicative phase, with EBV DNA copies being generated by replicating infected B cells.

## Other interventions

Owing to the potential serious impact of graft loss or patient death that can occur with PTLD, many investigators have attempted immunosuppression minimization as part of a broader attempt to reduce the frequency of post-transplantation infections, including BK virus nephropathy, CMV infection and transplant pyelonephritis. Such minimization can involve any of several strategies, such as calcineurin inhibitor dose and target level reduction, avoidance of induction antibody, and avoidance of steroids or anti-metabolites [42, 43]. Some interventions could be implemented in “pre-emptive” fashion, as discussed below.

## Prevention

Preventing PTLD is far more preferable to having to treat established disease. Primary prevention could be achieved by immunizing children against EBV prior to their becoming immunosuppressed. A vaccine developed against the gp350 envelope glycoprotein is currently in phase I/II clinical trials in the UK [7].

Secondary prevention has been tried by several investigators, using either serial peripheral blood EBV PCR monitoring or immune function monitoring. When EBV loads are seen to rise, immunosuppression drug levels are reduced, or specific drugs are discontinued, to reduce the total intensity of immunosuppression. However, in children, not all rises in EBV viral load necessarily lead to PTLD. Some children may develop a chronic high load carrier state [44], so the efficacy of this strategy is still not fully defined. Another form of secondary prevention is the use of anti-viral prophylaxis. The use of anti-viral agents has been discussed in the treatment section. As with treatment, use of anti-viral agents in prevention is also controversial, since the drugs do not prevent viremia from B cell proliferation. Both serial EBV monitoring and anti-viral prophylaxis have been associated with reduced incidence of PTLD, albeit in studies that were not randomized or prospective in nature.

## Outcomes

Though many cases of PTLD respond to therapy, some do not. The most recent data from a large pediatric kidney transplant multi-center trial suggest that there is an improved prognosis compared to the prognosis before [17]. Nevertheless, data from NAPRTCS indicate that the risk of graft loss is clearly higher after PTLD has occurred. The optimum time to restart high-dose immunosuppression is not known. For organs such as heart and lung, graft dysfunction or rejection can be immediately life-threatening. If graft loss has occurred, PTLD has not been reported so far in the re-transplants [45].

**Questions** (Answers appear after the reference list)

1. Which of the following interventions is *not* a preventive strategy for PTLD?
  - Reduction of immunosuppression
  - Anti-viral agent usage
  - Cyclophosphamide usage
  - Serial peripheral blood EBV PCR monitoring
2. Which of the following interventions is *not* a treatment strategy for PTLD?
  - Reduction of immunosuppression
  - Anti-viral agent usage
  - Cyclophosphamide usage
  - Serial peripheral blood EBV PCR monitoring
3. Which area of involvement with PTLD is associated with the worst prognosis?
  - Allograft involvement
  - Lymph node involvement
  - Central nervous system involvement
  - Bone marrow involvement
4. The following are risk factors for the development of PTLD *except*
  - Caucasian race
  - Recipient is seronegative for EBV at the time of transplant
  - Development of CMV disease
  - Use of mycophenolate mofetil (MMF)
5. Which of the following factors has the *strongest* association with PTLD?
  - EBV infection
  - CMV infection
  - BK virus infection
  - Urinary tract infection
6. In an immunocompetent host, the regulation and control of proliferation of the EBV-infected B cells occur primarily by means of
  - Plasma cells
  - Phagocytes (neutrophils, macrophages, dendritic cells)
  - Cytotoxic T cells
  - The complement system
7. In pediatric kidney transplant recipients most cases of PTLD are observed
  - Within the first year of transplantation
  - Between the first and second year after transplantation
  - Five years after transplantation
  - Immediately after treatment for acute rejection
8. All of the following statements are true, *except*
  - EBV viral loads may be elevated in the absence of clinical evidence of EBV disease or PTLD
  - EBV PCR is a useful screening test with high specificity for EBV disease and can replace histologic examination when the diagnosis of PTLD is considered
  - Immunosuppressive agents may result in false negative results of EBV serologic tests, even at the time of active EBV disease
  - Not every patient with PTLD will have elevated EBV viral loads

## Abbreviations

PTLD: Post-transplant lymphoproliferative disease
EBV: Epstein–Barr virus
CMV: Cytomegalovirus
PCR: Polymerase chain reaction
CsA: Cyclosporine A
CTL: Cytotoxic T lymphocytes
